# Discharge of a Fœtus through the Rectum

**Published:** 1868-11

**Authors:** 


### Discharge of a Fœtus through the Rectum.

Dr. Koehler exhibited to the N. Y. Pathological Society, the skeleton of a fœtus that had been passed per rectum. A lady, twenty-four years of age, became pregnant for the second time. The first three months of the pregnancy were passed under continual hypogastric pains. Then, suddenly, a pint of coagulated blood escaped through the vagina, whereupon the pains decreased and discontinued. She went to a physician well known to me for advice. When he tried to introduce the uterine sound into the orifice, he was unable to succeed. The cervical portion of the uterus was scarcely accessible. The patient consulted several other physicians, who advised her to await events. Normal movements of the fœtus from the end of the fourth month to the end of the pregnancy were ascertained. The prolimina of the birth appeared at the right time. The pains, however, had no effect; they lasted for three weeks, decreased by and by, and finally subsided. Then the secretion of milk took place. The patient became emaciated and cachectic. Two months after the end of the normal duration of pregnancy, rectitis and an abscess in the anterior wall of the rectum made their appearance, and a quantity of decomposed pus and ichor soon escaped through the rectum. Hairs of a fœtus were detected in the discharged matter. The skeleton of the fœtus then escaped through the rectum within the period of three days. The bones of the cranium following, the other bones were removed by the means of a polypus-forceps, either entire or broken. The aperture of the abscess was located one and a half inches above the anus. The diameter of the opening, when relaxed, measured one inch. One month after the evacuation and removal of the bones, perfect convalescence and menstruation took place.

The enlargement of the abdomen during the whole period of the pregnancy was uniform, not lateral, and the cervix uteri, even at the end of that period, was for a closer examination inaccessible. The patient was not confined to bed.—*Med. Record.*

